# An Iterative Jackknife Approach for Assessing Reliability and Power of fMRI Group Analyses

**DOI:** 10.1371/journal.pone.0035578

**Published:** 2012-04-17

**Authors:** Marko Wilke

**Affiliations:** 1 Department of Pediatric Neurology and Developmental Medicine, Children's Hospital, University of Tübingen, Tübingen, Germany; 2 Experimental Pediatric Neuroimaging, Children's Hospital and Department of Neuroradiology, University of Tübingen, Tübingen, Germany; UCSF, United States of America

## Abstract

For functional magnetic resonance imaging (fMRI) group activation maps, so-called second-level random effect approaches are commonly used, which are intended to be generalizable to the population as a whole. However, reliability of a certain activation focus as a function of group composition or group size cannot directly be deduced from such maps. This question is of particular relevance when examining smaller groups (<20–27 subjects). The approach presented here tries to address this issue by iteratively excluding each subject from a group study and presenting the overlap of the resulting (reduced) second-level maps in a group percent overlap map. This allows to judge where activation is reliable even upon excluding one, two, or three (or more) subjects, thereby also demonstrating the inherent variability that is still present in second-level analyses. Moreover, when progressively decreasing group size, foci of activation will become smaller and/or disappear; hence, the group size at which a given activation disappears can be considered to reflect the power necessary to detect this particular activation. Systematically exploiting this effect allows to rank clusters according to their observable effect size. The approach is tested using different scenarios from a recent fMRI study (children performing a “dual-use” fMRI task, n = 39), and the implications of this approach are discussed.

## Introduction

Functional magnetic resonance imaging (fMRI) is based on the intrinsic contrast of oxygenated versus de-oxygenated blood. Using appropriate imaging sequences, this effect is observable in the so-called blood-oxygenation level dependent effect (BOLD-fMRI; [1]), which is now widely used in neuroscience research to detect brain activations.

One common approach to statistical analysis of fMRI-data is employing the general linear model [2] whereby statistical parametrical maps can be generated from the imaging data that allow drawing inferences on different levels. Single subject analyses typically represent the first level, allowing to assess the pattern of significant activation in this subject alone. This may be perfectly appropriate for single case studies, but one of the main drawbacks is that the statistical comparison of a single subject with a control group is highly problematic [3], [4].

One step further is the joint assessment of a small group of subjects, termed fixed-effects analysis. This approach only allows to assess the “typical” activation pattern in this group [5], [6]; due to the strong influence of single subjects on the resulting group activation maps, no inference above and beyond the particular group of subjects in this analyses can be made. Another approach is to perform conjunction-analyses [7], where the question of “joint activation” between individuals can be posed in different ways [8], [9], but again, results from a small group cannot be generalized.

In order to find such “average” activation patterns, allowing to extrapolate imaging findings from a group under study to the general population [5], [10], so-called random effects analyses are now commonly used, representing a second-level analysis. Here, parameter estimates from several subject's first-level analyses are taken “to the next level” where they are then jointly analyzed. In order for this to work, a certain minimum group size requirement must be met; classically, group sizes of at least 12 have been deemed sufficient [6]. However, the reproducibility of activations was reported to be poor in groups of 20 subjects each [11], and substantial variability of activation patterns can still be observed as a function of group size and composition [12], suggesting that larger groups may be required for reliable (stable) results. However, such reliability is not easily inferred from group results.

In order to assess a given random-effect group map, it would be interesting to see the reliability of activation when systematically altering group size and/or composition. In this manuscript, “reliability” is used in the sense that it indicates whether activation in a voxel can still be detected if the group composition is altered. Clearly, an activation focus that disappears from such a map upon the exclusion of one subject must be interpreted more cautiously than an activation that remains significant even when excluding several subjects. This must be expected to be particularly relevant in the setting of an inhomogeneous group [3], [13] as the rate of change will depend upon the group homogeneity. Moreover, the group size upon which an activation focus disappears must be expected to reflect the “power” of this activation insofar as this number reflects the minimum group size required to detect this activation. In this manuscript, “stability” is used in the sense that it indicates whether activation in a voxel can still be detected if a smaller group is assessed.

With this manuscript, an approach is put forward that is aimed at addressing the reliability of fMRI activation on the group level by iteratively re-analyzing a given group, following the systematic removal of one or more subjects from it. This approach results in multiple, instead of one, group activation maps, the overlap of which can be taken to be reliable even in the context of slightly smaller and/or differently-composed subgroups. The concept as well as the implementation shall now be described in more detail.

## Methods

### General Approach

The basic idea is to generate several subgroups from a given group of subjects (contributing to a given second-level analysis). For example, by removing a single subject from a design with *n* subjects, a new reduced analysis with *n*−1 subjects ensues. While certain differences must be expected to be present between those two analyses due to loss of power alone [12], the overall activation pattern (which, in both cases, is interpreted to be generalizable to be the average activation pattern of the general population [5]) should be similar. This “similarity” is assessed in a systematic fashion here by iteratively removing every single subject in a first step and then every possible combination of 2, 3, or more subjects (see below for computational limits). This constitutes an iterative jackknife approach, which again is a special form of the bootstrap [14]. However, a bootstrap explicitly samples with replacement [15], which does not make sense here. A similar approach was recently suggested in the context of functional localizers [16] and was used earlier in the determination of reliability of single-subject activations [17].

In order to assess the reliability of activations, results from the reduced analyses are combined in order to identify areas of overlapping significant activation. This, in a simplistic way, constitutes “a new level” of analyzing fMRI group data, tentatively termed “third level”, L3 (in analogy to single-subject [L1] and group [L2] analyses [2], [5], [10]). A convention is suggested that an activation pattern can be considered “very reliable” if it is present in all reduced analyses (100%), which is the approach used here and before [16]. Results could still be considered “reliable” if they are present in the majority of reduced analyses (>50%). Activations present in less than half of reduced analyses (<50%) must be considered “unreliable” in this context; interpretation of such activation may have to be more cautious. A single descriptor can be used, such as 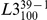, describing the “very reliable” third level results from an original design of *n* = 39 from which one subject was iteratively removed.

When iteratively removing subjects, activation foci will start to disappear as the detection power of a design with fewer subjects is reduced [18]. This effect can be used to indirectly assess the strength of the underlying activation as a “stable” activation will be detectable even in a design with fewer subjects. Conversely, an activation that only becomes significant when including more subjects is likely “unstable”. While this minimum number of subjects cannot be routinely inferred from a given second-level group map, the approach here can be extended to do just that, by iteratively removing subjects (up to a pre-specified minimum, set to 12 here [6]). This allows detecting the minimal group size that is necessary for a given focus of activation to become significant. In effect, this constitutes a post-hoc power analysis, assessing the observable effect size [19]. Jointly assessing significance and effect size allows being more confident about the validity of the conclusions that are drawn from the results.

### Implementation

The algorithm is implemented within the SPM8 software environment (Wellcome Department of Imaging Neuroscience, University College London, UK) and was developed using Matlab R2011a (The Mathworks, Natick, MA, USA). The user has to interactively specify the required inputs (parameter maps, covariates, and number of subjects to remove), upon which the original as well as the reduced designs are calculated (alternatively, inputs can be passed via the command line).

In the simplest case of removing one subject, there will be *n* analyses to perform (in our example [see below] of 39 children, there will be 39 reduced analyses with 38 subjects each). However, the number of possible unique combinations (as removing subjects X and Y is equivalent to removing subjects Y and X) is determined according to
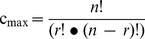
(1)with c_max_ being the maximum number of combinations, *n* being the original number of subjects, and *r* being the number of subjects to remove. It is obvious that this quickly results in an unfeasible number of possible group analyses (for example, when removing 9/39 subjects, there are 1.67 * 10^9^ combinations), which makes it necessary to limit c_max_. For the purpose of this manuscript, a maximum number of 100 group analyses was calculated for each step, randomly selected from all possible combinations. This number seems sufficient and additionally ensures that, for the group percent overlap maps, each reduced analysis contributes 1%. In order to assess whether this results in a lack of accuracy, each scenario (see below) was also calculated using a maximum number of 1000 group analyses, and results were compared using the Mann-Whitney U-test, with significance assumed at *p*≤.05, Bonferroni-corrected for multiple comparisons.

Following estimation of the reduced design, t-maps are generated by applying the appropriate (user-defined) contrast, which are then thresholded at a given level of significance (either using no or the family-wise or FDR-approach to correcting for multiple comparisons [20], [21]). Each map is compared to the t-map from the original design, using an indicator of spatial overlap, the Dice similarity index. This index is calculated according to
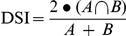
such that Dice's similarity index is calculated as twice the sum of overlapping significant voxels between to images A and B, divided by the sum of significant voxels in both images. This index ranges from 1 (perfect overlap) to 0 (no overlap), with values of .7–.8 being considered “high” [22]. It should be noted that zero overlap is also found when one reduced design fails to yield significant voxels. For each reduced design, one value is generated, resulting in 100 values per step. Additionally, all thresholded maps from each step are combined, resulting in a single image volume where the voxel value represents the overlap of significant activation (e.g., a voxel value of 75 indicates that this voxel is significant in 75% of all reduced analyses in this step, i.e. is “reliable”). This constitutes a group percent overlap map (gPOM), similar to approaches used previously [16], [23], [24].

### Imaging data

For the purpose of this paper, imaging data previously acquired from a group of healthy children was used, performing a “dual use” fMRI task that allows to investigate both language and visuospatial functions [25], resulting in two group analyses. Subjects were recruited from the general population; they were excluded due to general MR-contraindications as well as due to prematurity, neurological or psychiatric morbidity, or severe prior illness. Handedness was assessed using the Edinburgh handedness inventory (EHI [26]). The study was approved by the Ethics committee of Tübingen University Hospitals; all parents gave written informed consent, and all children gave assent prior to scanning. Overall, 39 children could be included, mean age 12.23±2.58 years, range, 7.9–17.8, 21 boys, 18 girls, EHI = .69±.47, range, −1 −1.

### MR-Imaging and data processing

Children were imaged on a 1.5T MR scanner (Siemens Avanto, Siemens Medizintechnik, Erlangen, Germany) with a standard 12-channel head coil. An EPI-sequence was used to acquire functional series in each subject (TR = 3000 ms, TE = 40 ms, 40 axial slices, yielding a voxel size of 3×3×3 mm^3^), covering the whole brain including the cerebellum. A T1-weighted anatomical 3D-dataset (176 contiguous sagittal slices, in-plane matrix 256×256, yielding a voxel size of 1×1×1 mm^3^) and a gradient-echo B0-fieldmap were also acquired. All processing and analyses steps were done using functionality available within SPM8, as described previously [25]. Briefly, images were initially subjected to a wavelet-based denoising scheme [27] and were motion-corrected in the next step, simultaneously removing EPI distortions and EPI*motion interaction effects [28], using the individually-acquired fieldmap. Subjects with translations exceeding voxel size (3 mm) in either direction were removed. The anatomical dataset was segmented [29] using custom-generated pediatric priors [30] and, following coregistration, the thus-derived spatial normalization parameters were applied to the functional images which were written out to a resolution of 3×3×3 mm^3^. Global image signal drifts were removed [31], and images were smoothed with a Gaussian filter of FWHM = 9 mm.

On the first (individual subject) level, statistical analysis was performed applying the framework of the general linear model [2], using a box-car reference function convolved with the hemodynamic response function. Applying the appropriate contrast, this resulted in contrast images which were then taken to the second level. Here, age (in months), gender, and handedness were considered confounders and were used as covariates of no interest [32], [33]. Significance was assumed at *p*≤.05, FWE-corrected for multiple comparisons, except when stated otherwise.

### Different scenarios

The approach was tested in different scenarios as follows: group size is one of the main determining factors for the stability of activations on the group level [12], suggesting that the overlap between the original and a reduced analysis (with *n*−1) should be higher in larger groups. To test this hypothesis, different group sizes were simulated, assessing the whole group of *n* = 39 as well as 29 and 19 subjects which were randomly selected from the whole group. Further, the overlap between similar groups must be expected to be a function of group homogeneity: in the presence of outliers [13], [24], overlap between maps including vs. not including the outlying subject must be expected to be smaller. This hypothesis was tested by including one intentional outlier into each group scenario, which was achieved by inverting the parameter map of one subject (which will lead to activation in parietal, not frontal, brain regions [25]). Finally, the overlap between images must be expected to be a function of the stringency of the applied thresholding approach: a stricter approach will eliminate more voxels, thus likely reducing overlap. This hypothesis was tested by comparing FWE- and FDR-approaches to accounting for multiple comparisons [20], [21].

## Results

### Standard second-level analyses

**Figure 1:**
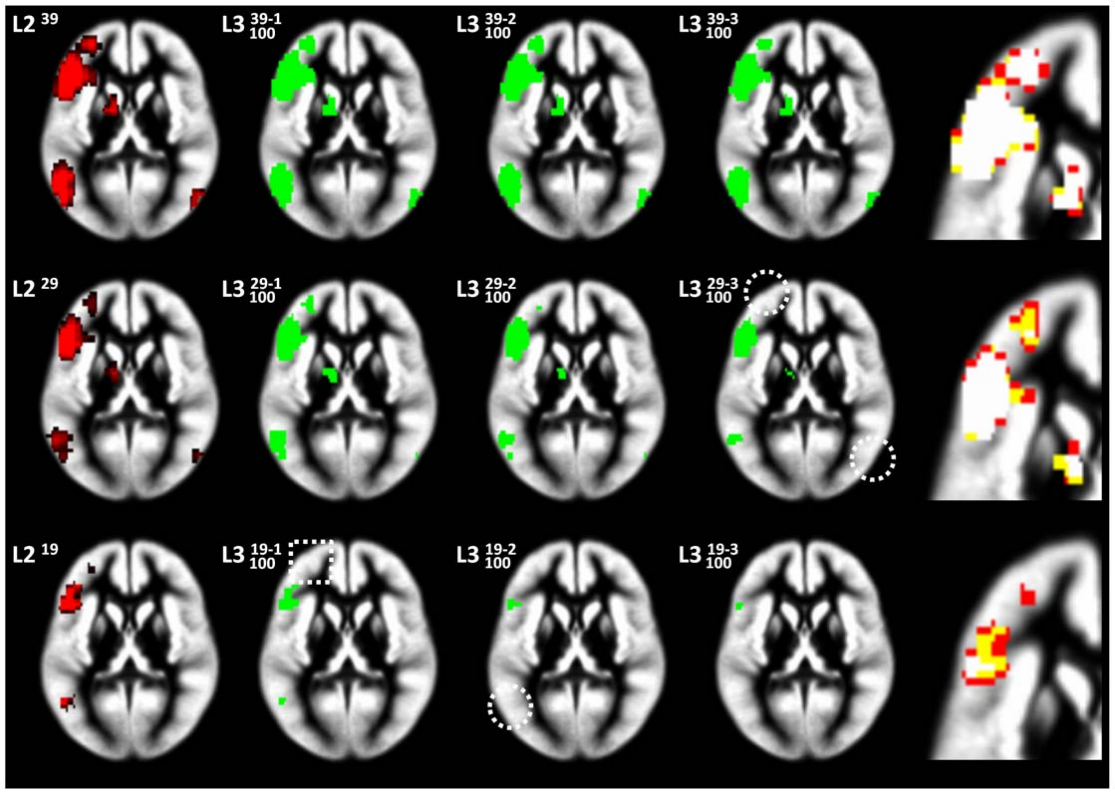
Language functions: standard second-level (L2) random effects as well as third-level (L3) analyses. L2 (left column) for three scenarios (*n* = 39 [top], 29 [middle], and 19 [bottom row]), and L3 (middle columns) following the removal of 1, 2, or three subjects. Right column: magnified overlap between the L3-maps: white voxels indicate overlap in all three maps. Note disappearance of smaller clusters in reduced analyses (white circles) due to loss of power and consecutively less overlap in L3-maps of the designs with fewer subjects.

**Figure 2:**
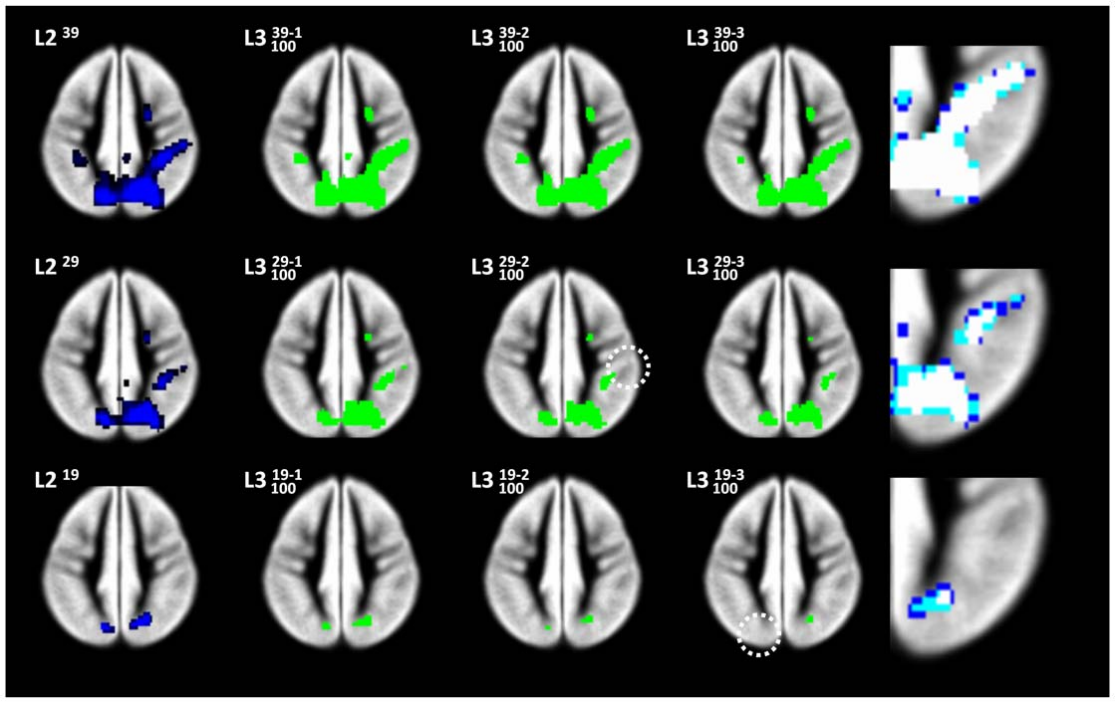
Visuospatial functions: standard second-level (L2) random effects as well as third-level (L3) analyses. L2 (left column) for three scenarios (*n* = 39 [top], 29 [middle], and 19 [bottom row]), and L3 (middle columns) following the removal of 1, 2, or three subjects. Right column: magnified overlap between the L3-maps: white voxels indicate overlap in all three maps. Note disappearance of smaller clusters in reduced analyses (white circles) due to loss of power and consecutively less overlap in L3-maps of the designs with fewer subjects.

The standard second-level analysis reveals activation in left-dominant inferior and middle-frontal as well as posterior-temporal language regions bilaterally for the language>visuospatial functions contrast (Figure 1, left column), and in posterior-parietal and high-frontal brain regions in the visuospatial functions>language contrast (Figure 2, left column). The effect of assessing the smaller groups with *n* = 29 and *n* = 19 is clearly visible in a reduction of detection power.

### Third-level analyses: group percent overlap maps

For the third-level extension to the standard second-level analysis, the effect of iteratively removing 1, 2, or 3 subjects from the three scenarios (with *n* = 39, 29, and 19 each) shows the major foci of activation unchanged: they are “very reliable” (Figure 1 & 2; middle panels). However, smaller foci are not reliably active in the reduced analyses (circles in Figures 1 & 2). The high reliability of the center of activation is clearly seen when directly assessing the overlap of the “reliable” voxels, as exemplified for the left inferior frontal cluster in the language > visuospatial functions contrast and the right-parietal cluster in the visuospatial functions > language contrast (Figure 1 & 2, right panels).

### Third-level analyses: Dice's similarity index

**Figure 3:**
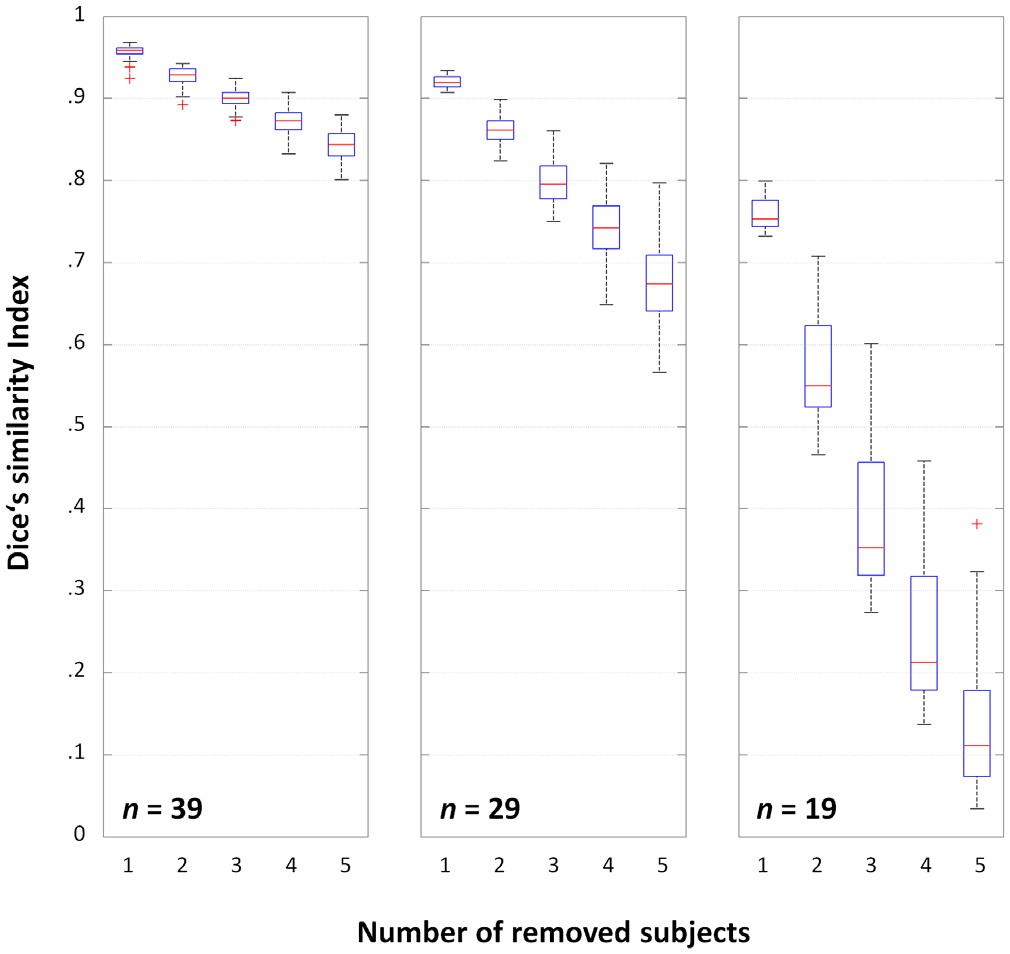
Dice's similarity index for the original and reduced designs, following exclusion of 1–5 subjects. Boxplots for each scenario (*n* = 39 [left], 29 [middle], and 19 [right]) show stronger decline in overlap as a function of group size, indicating more reliable activation in the larger group.

**Figure 4:**
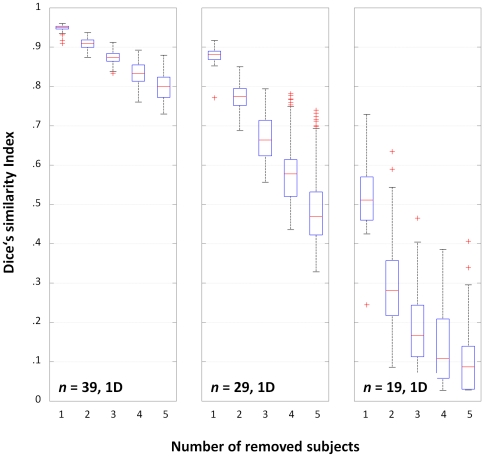
Dice's similarity index for the original and reduced designs with one deviant (1D), following exclusion of 1–5 subjects. Boxplots for each scenario (*n* = 39 [left], 29 [middle], and 19 [right]) show much stronger decline in overlap due to increased group inhomogeneity (cf. Figure 3). This is most pronounced in the smaller groups, indicating their higher vulnerability to outliers.

**Figure 5:**
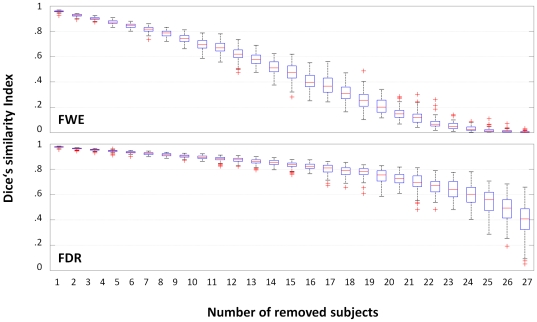
Boxplots of Dice's similarity index between the original (*n* = 39) and reduced designs as a function of thresholding approach. When excluding 1–27 subjects using the FWE- (favoring specificity) vs. the FDR-approach (favoring sensitivity) to controlling type-I-errors, there is a much stronger decline in overlap due to the stricter elimination of voxels in the FWE-approach, with DSI approaching 0 due to lack of significant activation in some analyses.

The DSI for removing 1, 2, or 3 subjects from the three scenarios (with *n* = 39, 29, and 19) are shown in Figure 3. As hypothesized, the effect of removing subjects is less pronounced when the group is larger. The effect of including one deviant (1D) in each group has the expected effect of reducing overlap between the original and the reduced design (Figure 4) and of increasing the variance, which again is more pronounced in the designs with fewer subjects (cf. Figure 3). When controlling for multiple comparisons using the FWE-approach (favoring specificity) as opposed to the FDR-approach (favoring sensitivity), a faster and more pronounced decline in overlap between consecutive steps can be seen (Figure 5). When assessing the effect of calculating a maximum of 100 vs. 1000 group analyses per step, there were no significant differences in any scenario, and the largest difference in median DSI was .02 for *n* = 39, .012 for *n* = 29, and .014 for *n* = 19.

### Post-hoc power analyses

**Figure 6:**
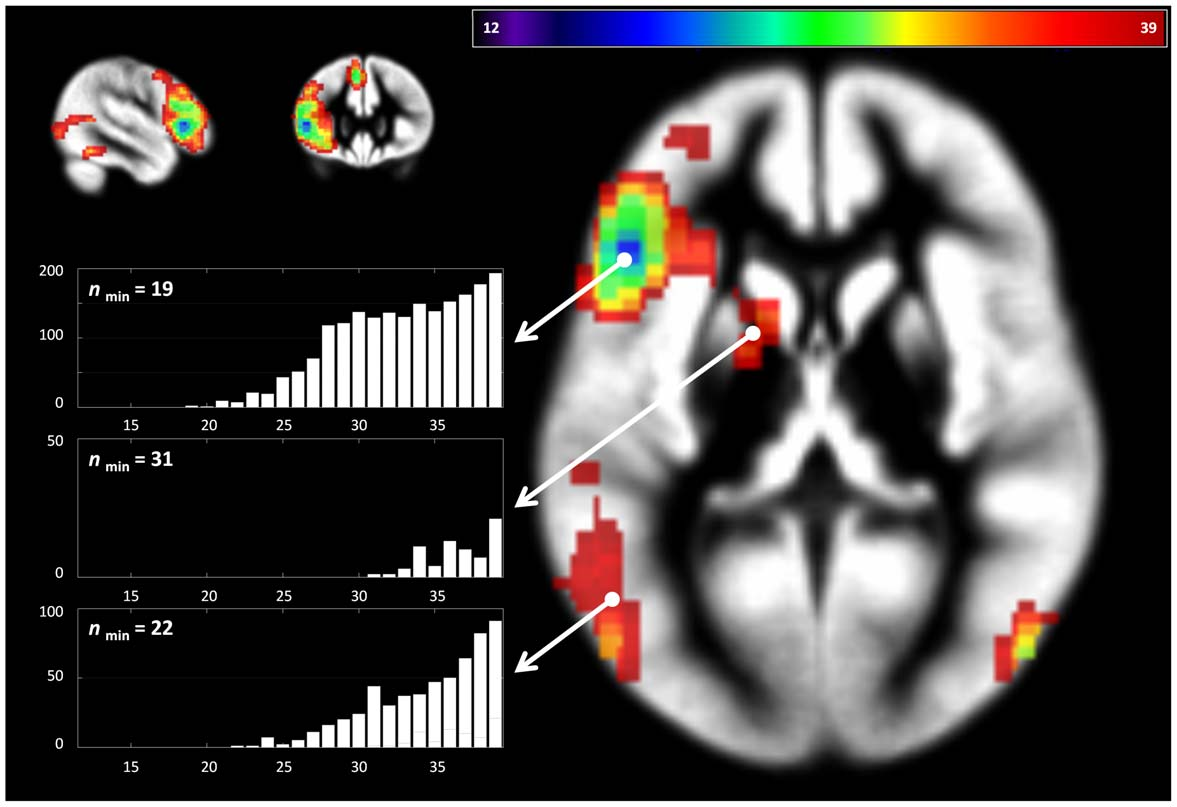
Language functions: results of post-hoc power analyses. This illustrates the minimum number of subjects required to safely detect a cluster (see text for details). Insert: plot of number of significant voxels (y) versus number of subjects (x) in the respective cluster (arrows). Note different minimum number of subjects required to detect a given cluster, allowing to rank results according to their observed effect size.

**Figure 7:**
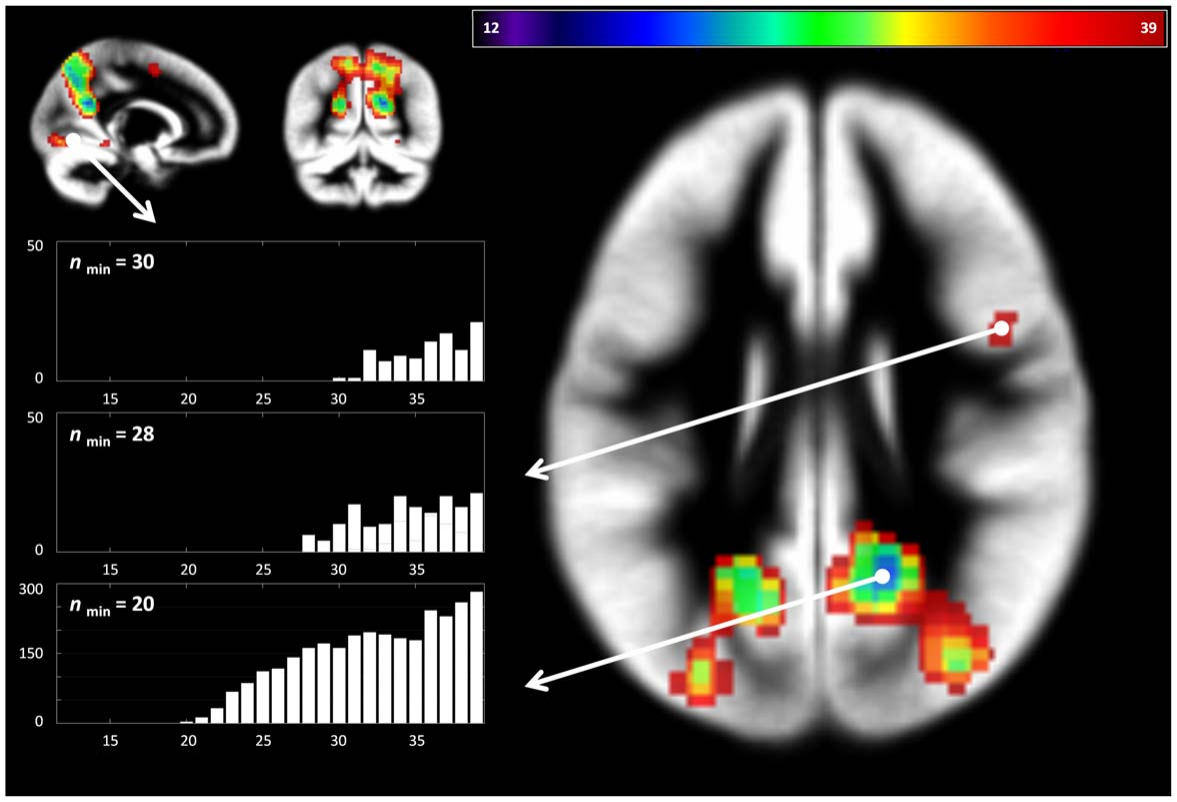
Visuospatial functions: results of post-hoc power analyses. This illustrates the minimum number of subjects required to safely detect a cluster (cf. Figure 6). Insert: plot of number of significant voxels (y) versus number of subjects (x) in the respective cluster (arrows). Note different minimum number of subjects required to detect a given cluster, allowing to rank results according to their observed effect size.

When assessing the minimum number of subjects that is required in order to detect a given cluster of activation, a clear hierarchy of clusters can be seen for each contrast (Figure 6 & 7). The major, “stable” clusters are safely detected with a smaller number of subjects, while the less stable, smaller clusters are only safely detected with a higher number of subjects.

## Discussion

In this work, a framework is suggested for assessing the reliability of functional activation patterns within a group. This can be extended to determine the observable effect size by performing systematic post-hoc power analyses. It is suggested that the assessment of the reliability of an activation as well as its observable effect size allows researchers to explore their results in more detail.

### Assessing reliability

Functional MRI group analyses will always change (more or less) when the contributing subjects change, as a function of the inherent and unavoidable group inhomogeneity, especially in smaller groups [11], [12]. Even though random effects analyses aim at being generalizable to the general population [5], [10], this remaining variability is a problem: it poses the dilemma that two analyses (say, one with *n* subjects and one with *n*-1), both of which should reflect the average activation pattern of the general population, may contradict each other (above and beyond the difference explained by the loss in detection power [18]). This is a problem as both analyses are equally legitimate. One way to deal with this dilemma is to increase group homogeneity by removing outliers/influential subjects [13], [24], [34]. As lower variance allows for the detection of smaller effect sizes, investigating a smaller, but more homogeneous group may be meaningful [35], [36]. While the identification of a single subject or a small number of subjects that “behave differently” can be done in a number of ways [13], [37], [38], the problem is to define what constitutes an outlier in the first place, and when it is justified to remove a subject. As already mentioned by Cook ([39], p15): “*the problem of determining which point(s) to delete can be very perplexing”*. This is particularly true in the absence of an obvious, plausible explanation: when identifying deviating subjects, the decision to remove them is made easy if their outlier status is explained by, e.g., technical problems or excessive motion [36], [40], and such datapoints should of course be identified and removed. However, if no such objective criteria exist, the subject may simply reflect an extreme manifestation of the normal range, e.g. due to using a different cognitive strategy [13], [41]. While rather narrow definitions of “normal” were suggested, rejecting 9/10 subjects [42], it is a matter of debate whether removing “unusual” subjects is always a good idea [37], [43] as a super-normal, artificially clean population may result (a problem known as “tidying-up bias” [44]). Moreover, an outlier usually constitutes “the most extreme subject” from a group; if it is removed, another subject will automatically become the next “most extreme subject”, making it difficult to draw a line on when to stop.

As an alternative to removing specific subjects in order to increase homogeneity, the approach taken here removes every subject instead in order to be less vulnerable in the presence of inhomogeneity. It is aimed at assessing the reliability of an activation pattern/focus in the context of a given group study by broadening the database. In other words, by systematically altering group composition, the results allow to infer not only “significance in this particular group of *n* subjects”, but also “significance in all (or most) possible subgroups of *n*–X”. According to the very first definition (“an analysis of analyses”; [45]), one could even refer to this as a kind of meta-analysis, but as most subjects will be present in most reduced analyses, the results are inherently not independent of each other [16]. The results inform the investigator with regard to how reproducible an activation pattern is when the original design is altered, making reliability transparent (as illustrated in Figure 1 & 2). In a simplistic way, this “third level” does not aim at addressing the between-session variance (as do second-level, random-effects analyses [10]), but instead addresses the variance between differently-composed subgroups by generating a readily-interpretable measure of concordance: the overlap of significant activations in all or most reduced second-level maps (with the limitation that, for computational reasons, by default only 100 reduced designs are calculated, see below). As can be seen from Figures 1 & 2, even smaller foci of activation are reliably detected in all reduced analyses until disappearing due to lack of power (see below). These smaller foci of activation can therefore be interpreted with a higher level of confidence than can be inferred from a single group map alone. Conversely, activation foci not present anymore in the majority of reduced analyses are confirmed not to be “very reliable” with the respective group size and/or composition (white circles and squares in Figures 1 & 2). Hence, important additional information above and beyond “significant (in *one* group) analysis” can be ascribed to every single voxel.

Such additional reliability is of course of major interest in experimental settings where the group size cannot easily be increased, as in special patient populations [40] or children [46]. Despite appreciating that activation patterns in fMRI group maps become more reliable when including at least 20–27 subjects [11], [12], this may simply not be feasible when only a limited number of “special” subjects is available. In such a setting, the ability to additionally demonstrate the reliability of a given group activation may allow for wider-ranging conclusions. Moreover, an assessment of reliability must be expected to show dramatic changes in group maps of more diverse populations, such as epilepsy patients with higher within-group variance [3], [47], potentially invalidating the use of parametric tests [13]. Consequently, the stepwise overlap in the original scenarios (Figure 3) is much higher than when including one deviating subject (Figure 4). Both figures also illustrate that group size is an important factor, and that group homogeneity is more important in scenarios with fewer subjects, as expected.

In comparison with previous studies [11], [12], the approach presented here does not aim at assessing the reproducibility of functional activations as a function of group size *per se* (although such effects can clearly be seen, cf. Figure 3). It is also not aimed at evaluating the reproducibility between repeated sessions [23], and is not used to resolve interdependence [16]. Also, in contrast to the approach taken by McNamee & Lazar [24], it is specifically not aimed at detecting outliers. Instead, each and every subject is removed, which is equivalent to a special form of the bootstrapping approach, called a jackknife [14], [15], applied iteratively. However, the sampling is not random but systematic; it is therefore more related to permutation tests already successfully used for analyzing neuroimaging data [48], [49]. Typically, the number of permutations is the limiting factor, which is also the case here: the number of possible combinations becomes prohibitively large (cf. equation 1), effectively requiring to undersample c_max_ (it is of course important to sample sufficiently, ensuring that every subject has an even chance of being removed). However, since the results from running 100 vs. 1000 reduced group analyses are not significantly different (and the effect size is small), the error associated with reducing the sampling rate to 100 seems negligible here. Using this sampling rate, exploring L3^39−1^, L3^39−2^, and L3^39−3^ required a total of 11 minutes on a current PC workstation.

### Power issues

**Figure 8:**
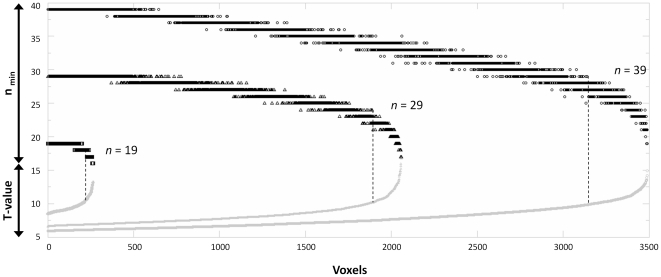
Comparison of the power indicated by post-hoc power analyses and t-values. For each significant voxel (sorted in ascending order on the x-axis), both the t-score (gray symbols) and the minimum number of subjects required for safe detection (black symbols) are plotted on the y-axis. Results are shown for three scenarios of *n* = 39 (circles), *n* = 29 (triangles), and *n* = 19 (squares). Note monotonous increase in t-values, but several corresponding minimum number of subjects, as well as different numbers of subjects corresponding to the same t-value in each scenario (*t* = 10; dotted black lines).

It must be remembered that a principal drawback of this approach is that all analyses on smaller groups are by default confounded by the issue of power: a smaller group will be less likely to detect group activations by this effect alone [12], [18], [50]. This is reflected in a decline of overlap when removing subjects (Figure 3), demonstrating that, by virtue of being less powerful, a smaller group of *n*−1 may not faithfully reproduce the significant results in the group with *n* subjects. However, if an activation is so barely above the detection threshold, this in and of itself is important information as it is obviously not very reliable. Moreover, this effect can actually be used to extend the concept described above to systematically analyze each and every voxel with regard to the minimum number of subjects required for it to reach significance. This is achieved by removing more and more subjects until a pre-specified minimum number is reached (default: 12 [6]). In effect, this constitutes a post-hoc power analysis, assessing the observable effect size [19]. Of note, the illegitimate use of such analyses, referred to as the “power approach paradox” [51], is not an issue here as results are only computed for voxels that are significant in the first place. Power analyses are as yet underrepresented in neuroimaging research, partly due to the issues with spatially different variances and temporal autocorrelation [50], [52], [53], [54] and the difficulty in defining the required effect size *a priori*; i.e., it remains problematic to predict beforehand how many subjects *will be* necessary to detect a given activation. The idea behind the extension presented here is to enable a researcher to assess how many subjects *were* necessary to safely detect a given effect, such as a cluster of activation in a given brain region, which may potentially be used as a reference for future studies. This number will of course depend on a number of factors [19], among them the statistical threshold used to control for type I-errors: a stricter approach (such as FWE) will require more subjects than an approach favoring sensitivity (such as FDR; see Figure 5). In effect, the minimum number of subjects can be ascribed to every significant voxel, and thus, every cluster (see Figures 6 & 7). This allows ranking the clusters as to their respective observable effect sizes, allowing to better understand the activation pattern seen in a given group. Of course, it could be argued that this information is also reflected in the magnitude of the resulting t-statistics, but this value is dependent on the degrees of freedom and can therefore not easily be compared between scenarios. For example, for a given t-value (e.g., T = 10), the corresponding minimal number of subjects is either 17 or 18 in the *n* = 19 scenario, ranges from 21–24 in the scenario with *n* = 29, and from 25–29 in the scenario with *n* = 39 (see Figure 8), also demonstrating that the correspondence between observable effect size and t-value is not unique. This exemplifies that the minimal number of subjects is a more direct, readily interpretable, and less ambiguous indicator, and it is suggested here that this metric may be a helpful indicator for characterizing observable effect size in functional MRI group studies.

### Possible limitations of this approach

It could be argued that the lack of formal statistical analysis of the multiple second-level maps is a major limitation: for example, extending the concept of conjunction analyses [7], [8] to the current setting might allow for more formal inferences to be drawn. Alternatively, different measures used to assess reproducibility (both between sessions and between sites), including intraclass correlation coefficients, coefficients of variation, Fisher's combining method, or kappa, among others [12], [23], [24], [55], [56], could be employed to assess “overlapping activation” in a more formal way. However, the simplicity of the approach could also be seen as its main advantage, conveying readily understandable information about this particular scenario under investigation.

**Figure 9:**
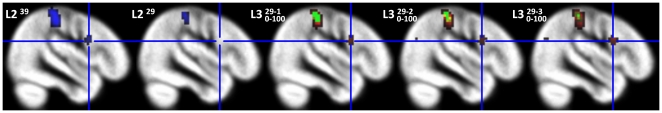
Illustration of “unreliable” activation potentially guiding data exploration. A significant activation in the larger group (L2^39^) is not seen in the smaller group (L2^29^, crosshair) but is detected as an “unreliable” activation in all three reduced analyses (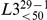, 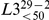, 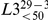). “Unreliable” activation (<50%) is shown in red, “reliable” activation (50–99%) is shown in yellow, and “very reliable” activation (100%) is shown in green.

The convention used throughout this manuscript is that a voxel is only considered “reliably active” if it is detected in all (up to 100) reduced designs, and is discounted if this is not the case. It must be admitted that this convention is clear, but arbitrary. Although the assessment in multiple reduced designs increases the available data base, requiring 100% may be overly strict, and other cutoffs might be equally justifiable, such as the assessment of activation present in more than half reduced analyses (“reliable”), or even the exploration of activation present in less than half reduced analyses (“unreliable”), as activation patterns only present in some analyses may guide further data exploration. An example is shown in Figure 9, where a significant activation in the larger group (L2^39^) is not seen in the smaller group (L2^29^) but is detected as an “unreliable” activation in all three reduced analyses (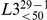, 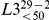, 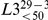). The argument is similarly valid for the power analyses, where the required overlap could be set to 80%, a threshold commonly used in power analyses [19]. Hence, further research is necessary to define the optimal threshold for different scenarios, for either application. Finally, similar approaches applied here to the voxel-level could be employed to assess reliability on the cluster-level; however, this would not be straightforward due to the non-stationarity of local smoothness estimates [57].

### Conclusions

To conclude, the approach presented here allows assessing the reliability (or lack thereof) of functional activation foci in group activation maps. This “third level” of statistical analysis may prove to be helpful especially in analyses of smaller groups and in settings with high intra-group variance. Post-hoc power analyses allow to rank clusters according to their observable effect size (stability) and enable the researcher to identify the minimum number of subjects that is required to detect a given activation.
